# Managing Thermal Injuries of the Penis and Scrotum: A Narrative Review

**DOI:** 10.3390/ebj4020016

**Published:** 2023-04-26

**Authors:** Tannon Tople, Alexander Skokan, Russell Ettinger, Shane Morrison

**Affiliations:** 1 School of Medicine, University of Minnesota Twin Cities, Minneapolis, MN 55455, USA; 2 Department of Urology, University of Washington Medical Center, Seattle, WA 98104, USA; 3 Division of Plastic Surgery, Department of Surgery, University of Washington Medical Center, Seattle, WA 98104, USA

**Keywords:** genitals, penis, scrotum, burns, frostbite, management, quality of life

## Abstract

While thermal injuries to the external genitalia occur less often than burns to the trunk and extremities, such injuries can potentially leave patients with devastating lifelong sequelae. Though much is known about treating burns in commonly exposed areas of the body, there is a lack of agreement concerning the management of genital thermal injuries. In this review, we seek to synthesize the past and existing literature into a clear analysis while reviewing current recommendations and new developments in the management of genital thermal injuries of the penis and scrotum. Specifically, recommendations for managing genital burns are discussed, including the role of urinary and fecal diversion, debridement, use of skin grafts, and flap coverage choice. Finally, less common thermal injuries, such as frostbite of the genitalia, are discussed.

## 1. Introduction

An estimated 486,000 individuals present to emergency departments in the United States seeking medical attention for all burn-related injuries each year [1]. Although less common than burns occurring on the trunk, extremities, and head, due to the region’s limited surface area and relatively protected anatomical location between the thighs, the incidence rates for burns affecting the genitalia are less pronounced and range between 1.7% and 16.9% of all burn injuries, though this may be an overestimation based on currently available data [2,3,4]. Likewise, frostbite injuries of the genitals, though rare, may also potentially contribute to the prevalence of thermal injuries needing medical treatment. Though people are unlikely to have a genital thermal injury, these injuries significantly threaten long-term physical, sexual, psychosocial, and psychological health.

For these reasons, it is crucial to explore the current management of thermal injuries affecting the genitals in the medical literature so that long-term sequelae may be prevented [5]. Yet, while much is known about treating burns covering more commonly exposed areas of the body, there is limited consensus in the literature regarding managing burns of the penis and scrotum. In this narrative review, we provide an updated review of how genital thermal injuries are managed so that practitioners may be adequately informed on how to treat patients and help them avoid debilitating health consequences.

## 2. Materials and Methods

A brief literature review was conducted utilizing Google Scholar, PubMed, and Medline. Articles were identified using search terms “penis” and “burns”, “perineum” and “burns”, “scrotum” and “burns”, “perineum” and “burns”, “penis” and “frostbite”, and “perineum” and “frostbite”. Identified articles were reviewed to confirm relevance to the subject of this review. No search restrictions were applied. Searches for frostbite were included in this review because this paper aims to include a broad summary of managing both types of thermal injuries of the penis and scrotum.

## 3. Discussion

### 3.1. Epidemiology

The epidemiology of burns may differ by region and sex. In the United States, most reported burns occur in males for each age group, except for those >80 years old [6]. Furthermore, like gender disparities, burns are disproportionately caused by several different mechanisms [6]. The leading mechanisms of overall burn-related injury for adults and children in the U.S. include fire/flame burns, scalds, and hot object injuries, accounting for 41%, 33%, and 9% of the total burn injuries reported, respectively [6]. Comparatively, some European countries may see more adult women than men who experience scalds and contact burns, emphasizing differences in burn-related epidemiology across geographic regions and cultures [7,8].

In the U.S., the prevalence of burns in men mirrors similar estimates about burn rates of the genitalia. Individuals with a penis and scrotum are at greater risk of developing burns on their genitalia, which is three times higher for males compared to females across all age groups [2,9]. Unfortunately, in European countries, there is a paucity of epidemiological evidence evaluating differences in genital thermal injuries based on sex. The increased prevalence in males may be attributed to anatomical differences, such as the larger surface area and more protuberant nature of the male genitals, making the penis and scrotum more vulnerable to thermal injury. Data show that isolated burns to the genitalia in all age groups most commonly occur on the penis and scrotum, accounting for 48.6% of cases, compared to 22.1% on the vulva/vagina [9].

Burns in the United States also exhibit a disparate impact on distinct age groups [2]. A significant proportion of all types of thermal injuries (22.8%) occur in children aged 0 to 9 years, which is nearly double the incidence rate of any other age group [2]. Unfortunately, this age group also represents the highest number of genital burn cases compared to other age groups [9]. In children (1 month–17 years), scalds accounted for the majority of genital burns (64.1%), then followed by flames (29.5%) [2]. This means that children are at a greater risk of thermal injuries to the genitalia, increasing the risk of long-term sequelae and morbidity from a genital burn.

Most genital burns tend to be considered severe, as second-degree (32.5–69%) and third-degree (28–67.5%) burns are most frequently reported [2,4,9]. This severity may be because genital burns are seldom found in isolation and typically occur alongside injuries affecting large surface areas [2,10]. Consequently, extensive burns involving the genitals are more likely to be fatal, and the involvement of the genitalia may be a predictor of fatality [4,10]. Nonetheless, mortality linked to the genital’s involvement in a burn is less probable to cause death in these patients. It is more likely due to complications from the extensive burn that also encompasses the genitalia [10].

Taken together, these data suggest that severe genital thermal injuries predominantly affect young individuals with a penis and scrotum. Given their younger age, it is essential to take extra precautions to minimize the risk of long-term sequelae in this patient population.

### 3.2. Anatomic Considerations

To establish the best approach for treating thermal injuries, it is essential to recognize the distinctions in skin pathology between various regions of the body. The skin in the genital area, similar to that of the hands, is thin, and contains fewer subcutaneous tissues than the torso and limbs [11]. As a result, the likelihood of more severe burns due to deeper tissue damage is higher [2,11]. Nevertheless, the genital area differs from most body parts in that it possesses an extensive vascular network supplying the skin and superficial subcutaneous tissues. This rich vascularization may suggest that the genital region has superior wound-healing capacity compared to other body areas. Overall, this understanding of genital tissue characteristics is important to consider as it often guides the management and approach taken for patients with genital burns.

### 3.3. Acute Management of Genital Burns

A thorough evaluation of the burn severity, the extent of injury, and the mechanism of the burn injury is the first fundamental component of devising an effective acute therapeutic strategy. The American Burn Association recently updated its guidelines in 2022 that describe the indications for initiating burn consultations and/or transfers to major burn centers [12]. A component of this criteria necessitates accurately classifying the burn’s degree [13]. Specifically, a first-degree burn solely affects the epidermis and may manifest on the patient as erythema and swelling, while a second-degree (partial thickness) burn involves both the epidermis and dermis (superficial or deep) and presents like a first-degree burn but with the development of blisters. Lastly, a third-degree (full thickness) burn involves damage to all skin layers, including the subcutaneous tissue, and presents as charred, waxy, and white. The patient may also experience a loss of sensation in affected regions. Irrespective of the burn’s surface area, third-degree burns and groin burns are categorized as “major burns” and necessitate escalated medical care. Although it may be challenging to distinguish between the different burn degrees clinically, patient management frequently depends on the burn’s severity, as discussed below and throughout this review. Developing the ability to identify burn severity correctly is thus vital to the acute management of genital burns.

If the genital burn is categorized as a first-degree or superficial second-degree burn, a conservative approach to treating the regions should be considered for most cases. Conservative management of genital and perineal burns is pursued because it has been shown to be highly successful in the acute setting. Specifically, conservative management is kept relatively straightforward and consists of wound changes and topical antibiotics such as 1% silver sulfadiazine (a topical antibiotic) as needed. Successful management of genital burns using these methods has been well-described in a 14-year study evaluating the management of perineal and genital burns. In this study, 87% of patients with genital or perineal burns required only topical antibiotics and local wound management [14]. A separate study also found similar results after reporting that 96% of genital burns had healed without surgical intervention or skin grafting [10]. These findings underscore the importance of recognizing the severity of burns and selecting appropriate management strategies, as the high patient recovery rates rely heavily on receiving conservative management. On the other hand, recognizing severe burns is also vital for management as further interventions may be required if there is a delay in wound healing, the burn is complex, or classifies as third-degree [14,15].

### 3.4. Management of Deep-Dermal Partial or Full Thickness Burns

#### 3.4.1. Debridement

While conservative management is considered the gold standard for first-degree or superficial partial-thickness burns, there exists no consensus about several aspects of managing severe genital thermal injuries [16]. Specifically, one consideration in managing a patient with severe genital burns is to determine if the area needs to be debrided. After an area is burned, debridement aims to remove grossly nonviable tissues to facilitate wound healing, control superinfections, or remove burned eschar [14,15]. This practice has been a topic of disagreement in treating genital burns as problems with surgical debridement occur because of the lack of discernment between viable and non-viable tissues [13]. Additionally, wound healing outcomes may be no better compared to conservative management, depending on the area that was debrided [17]. Because of this, several researchers have treated both simple and severe burns conservatively and without debridement [14]. Others have adopted a wait-and-watch approach, where they believe it is beneficial to only observe the wound closely for how necrotic vs. viable tissues evolve while performing local wound care over the initial post-injury period [14].

If debridement is pursued, the timing of when the burned area is debrided has also been debated. Specifically, there is disagreement about conservative vs. early or late debridement of a genital burn. Conservative management for treating deep-dermal burns may be effective due to the area’s high vascularity of genital tissue and, subsequently, higher recovery rates, as discussed previously. This has led some practitioners to believe that even some severe burns do not need to undergo radical debridement [14,18].

On the other hand, early debridement is successful for severely burned genitalia in children, as younger ages indicate a greater propensity for healing [19]. For example, in a report of eight young patients who underwent early debridement of genital burns and full-thickness skin grafting, all patients had excellent outcomes with the return of sexual function later in life [11]. Success was attributed to early debridement because the severity of thermal injuries is typically full depth, so little time was spent waiting to assess viable vs. non-viable tissues. Additionally, in eight adult patients who had experienced chemical burns of the penis, authors reported successfully treating them with early debridement and had satisfactory patient outcomes [11]. Successful management through both methods has been reported to have success, but the overall strength of these recommendations must be interpreted with caution as there has been limited scope in most reported studies.

If surgical debridement is not considered, there is potential to consider alternative forms of debridement, such as enzymes. The goal of enzymatic debridement compared to surgical is that practitioners want to optimize all viable tissues. In a single-center study, researchers began using enzymatic debridement in three patients. While long-term follow-up studies assessing outcomes of enzymatic debridement of the genitalia are undergoing, the authors noted “good results” after 12 months of follow-up in these three patients [20]. Based on these limited outcomes, the evidence for the efficacy of enzymatic debridement for genital burns is not highly recommended but remains a future area of investigation.

Overall, recommendations should include an individualized approach for each genital burn that considers burn severity, age, and progress in wound healing before undergoing surgical or enzymatic debridement. Treatment should also favor conservative management whenever possible.

#### 3.4.2. Skin Grafts

Though less frequent, skin grafts may be required for acute management for large defects, non-healing perineal wounds, or injury to the mons. Specifically, skin grafting is most likely to be used on deep second-degree or full-thickness burns of the penile shaft and scrotum, with full-thickness or split-thickness skin grafts used [21]. Furthermore, the application of skin grafts is not uncommon with genital burns. In a 20-year study assessing the management of pediatric genital burns, 50% required skin grafting [22]. Ultimately, the main indications for determining if a skin graft is needed vary but should be considered in burns that are full-thickness or partial-thickness burns that do not heal properly.

Several considerations need to be made when determining the type of skin graft to use for penile or scrotal coverage. If possible, patient expectations should be addressed prior to the use of a skin graft. Specifically, determining whether the patient intends to be sexually active may indicate the type of mesh technique that should be used. In patients who indicate they wish to continue being sexually active and have defects on their penile shaft, thick unmeshed split-thickness grafts skin grafts (0.04 mm; 0.0016 inches) may be preferable as they are more flexible, have better aesthetic outcomes, and would be less likely to result in secondary contractures [23]. These types of grafts are typically harvested from the medial thigh, applied to the penis, and secured with absorbable suture [23]. Unfortunately, the application of unmeshed grafts must be considered against the fact that they may be less likely to survive grafting [24]. For those less likely to be sexually active, meshed skin grafts applied to the penile shaft may reduce the risk of graft loss but run the risk of developing contractures, a common potential complication with long-term consequences and further operations [23].

Split-thickness skin grafts are also commonly used for scrotal reconstruction [24,25,26,27]. Though at a greater risk of contracture, this drawback may actually be advantageous when applied to the scrotum as it may improve the aesthetic appearance [26,28]. Likewise, split-thickness grafts have good outcomes, as demonstrated in a study (*n* = 27) where 17 (66%) adult patients achieved a “thin pliable scrotum,” and only 18% developed small adhesions when split-thickness grafts were applied [23]. Based on these results, the gold standard for both penile and scrotal reconstruction following a genital burn is split-thickness skin grafts.

Cadaveric cryopreserved allografts (CCA) may be used temporarily if a graft cannot be obtained from a patient [29,30]. The advantages of using allografts have ranged from improved wound healing to decreased pain associated with smaller donor graft sites and fewer wound changes [30]. Additionally, a type of allograft called dehydrated human amniotic/chorionic membranes (DHCAM) have been used in place of cryopreserved allografts where it is theorized that inherent growth factors in these tissues may improve fibroblast recruitment and wound healing. A small (*n* = 52) retrospective, single-center study found that compared to cadaveric cryopreserved allografts, adult patients with genital burns who received DHCAM needed significantly fewer reoperations [31]. Considering the study’s small sample size, these results should be approached with caution as their application in actual clinical settings is not fully supported or investigated. Nevertheless, the publication of this study illustrates the increasing number of novel technologies being used to manage genital thermal injuries.

Synthetic skin grafts are also used for genital burn injuries. Integra, an artificial skin created to treat second- and third-degree burns, has also been utilized in genital burns [32]. Typically, this type of skin graft is used if surgeons and patients wish to avoid hypertrophic scarring and wound contracture [33]. In a case series following three adult patients with full-thickness penile shaft burns, the application of Integra was effective. All three patients achieved a highly durable post-surgical outcome with an eventual return of sexual function, though some expectedly reduced sensation [22]. Because of this, synthetic skin grafts may also be considered when treating a genital burn.

All in all, it should be noted that CCA, DHCAM, and Integra may help aid in wound healing but are not considered complete alternatives to final wound closure for burns of the genitalia. This is because CCA and DHCAM are temporary, as they will inevitably be rejected from the body, and Integra requires a second operation.

#### 3.4.3. Flaps

For full-thickness genital burns, treatments for larger wound deficits may be needed. Allowing the area to close by secondary intention may lead to unnecessary contractures of the genital region, or if contractures do form following recovery of the genital burn, flaps may be considered [34]. Specifically, there are several flaps to choose from depending on the anatomy being reconstructed.

With regard to scrotal reconstruction, the size of the deficit needs to be considered. Some literature states that if <50% of the scrotum is affected, a scrotal advancement can be performed, while deficits greater than 50% of the scrotal surface area may require a flap from other areas [35,36]. One such example is the bilateral pedicled gracilis flap that may be used. Though considered controversial due to the potential risk of inhibiting spermatogenesis [15], other case studies have demonstrated good aesthetic outcomes and ease of use when covering severe deficits on the scrotum [37]. Though no long-term follow-up data are present and spermatogenesis has not been examined in people who received a gracilis flap, this intervention, in conjunction with split-thickness skin grafts, has been demonstrated to achieve high patient satisfaction, high flap survivability, and minimal complication rates (25–29%; including hematoma, abscess, venous congestion, and wound dehiscence; *n* = 20) [28,38,39,40].

Additionally, there has been success in scrotal reconstruction with neurovascular pedicled pudendal flaps. In two studies (*n* = 18), patients who received a pudendal flap for scrotal reconstruction achieved aesthetically satisfying outcomes while maintaining high flap survivability [38,39,41]. Furthermore, anterolateral/medial thigh flaps have been well-documented for covering the perineum and scrotum [42,43]. The anterolateral and anteromedial thigh flaps may be good options if the patient’s flap survivability is a priority. Specifically, anterolateral and anteromedial thigh flaps have been shown to have high survival rates at 100% (*n* = 20) and 95–100% (*n* = 40) in the literature, respectively [42,43]. Considerations for using an anteromedial flap must be weighed against a relatively high complication rate (24%) where tissue necrosis, fistula formation, infection, and flap breakdown have been reported [44]. If a flap is needed, there are several options for scrotal reconstructions.

Regarding penile reconstruction, there are several flaps to consider based on patient preferences. Specifically, a two-staged scrotal flap may be considered. This approach is particularly useful if only the penile shaft is affected. In a small study (*n* = 17) investigating the outcomes of this two-staged surgery, approximately 90% and 77% of adult patients achieved aesthetic satisfaction and had adequate erectile function, respectively, and only 17.6% experienced wound dehiscence as the primary complication [38]. The benefits of performing this procedure may be due to the flap’s lower complications with infection and flexible contexture, but staged procedures are difficult logistically for patients who are underserved and under-resourced [38].

If the penile injury is severe, a neophallus may be made. Depending on the patient’s preferences, phalloplasty may be pursued if the patient is younger, values having an aesthetic and sensate phallus, would like to pee in the standing position or is interested in engaging in sexual intercourse [45]. To achieve this, penile reconstruction may be completed with a radial forearm flap. Outcomes in males who have had phalloplasty have been positive, with reports of adequate satisfaction with appearance, tactile sensation, and coital function in several case reports and metanalyses [45,46,47]. Admittedly, complications for this procedure are high (76.5% overall; 34.1% with urethral fistulae and 25.4% with urethral strictures) and should be considered during clinical decision-making [45].

Overall, there are several advantages and potential disadvantages for selecting a flap that will cover a genital defect. The literature has reported that there is no clear preferred flap choice for genital thermal injuries of the penis or scrotum. Management and selection of a flap, if needed, must be made based on the patient’s unique injuries, surgeon’s preference, and other individualized circumstances [48].

### 3.5. Urinary and Fecal Diversion

Urinary diversion may also be needed in patients with genital burns, but there has been disagreement about the effectiveness of this intervention. In a study by Mcdougal et al., a high incidence of infectious complications in adults who underwent catheter drainage during postinjury burn care was reported. These complications included pyelonephritis, periurethral or prostatic abscesses, and suppurative epididymitis (67%) [3]. Following this study, urinary catheterization used in the management of genital burns was not recommended. Yet, several other studies have suggested that relatively few complications arise from catheterization [10,19].

Since these reports, there has still been mixed data regarding catheterization. In a study by Peck et al., 35% of their patients were not catheterized, treated with only conservative management, and still healed with good outcomes [10,19]. Furthermore, though less consequential than previous evidence had determined, others agreed that patients could heal mostly without catheterization. Follow-up studies from Peck et al. and an additional study by Michielsen et al. indicated that 41–50% of all burns to the genitals were successfully treated without urinary catheterization or diversion [10,14]. On the other hand, in a 10-year retrospective study by Angel et al. analyzing perineal burns in children, 66.7% required foley catheters, with UTIs reported as the most frequent complication [2]. In this study only severe urinary infections occurred, predominately in children under one year of age (17% of patients total) [2]. Altogether, there is moderate evidence to make recommendations about not requiring catheterization for genital burns, though these data should be carefully interpreted.

Today, most may recommend that urinary catheterization is not used for treating genital burns due to the inherent risk of nosocomial UTIs, but this remains controversial as these recommendations are based on data collected greater than a decade ago and rates of UTIs obtained from catheters are highly variable [10,19]. Additionally, avoiding catheterization in patients that can void spontaneously is highly encouraged. Thus, catheters should be clearly indicated for one purpose only: to monitor urine output and record strict intake/output for the purpose of initial patient resuscitation [10,19,49]. If a catheter is placed in a patient, the integrity of the urethra must be monitored and efforts should be made to remove it as soon as possible to prevent the development of urinary tract infections and urethral sloughing [50]. If the urethra is not capable of passing a foley, a suprapubic tube may be placed to monitor for resuscitation over time [49]. Though theoretically possible to help with urethral stricture formation, the literature has not examined the indwelling catheter’s capacity to reduce urethral strictures; instead, it is more likely to cause stricture formation [51,52].

While treating burns of the genitals and perineum, there is also a concern that fecal contamination of these wounds could lead to sepsis, graft loss, and UTIs [53]. Past recommendations for managing contamination have included diverting colostomies or fecal management systems (FMS) [53]. Since those recommendations, several studies investigating this intervention determined that diverting colostomies proved futile in preventing fecal contamination of burns and are thus unnecessary in managing genital burns. Though there is limited evidence that fecal management removal systems improved mortality outcomes (61% down to 25%) in admitted patients with perineal burns (*n* = 36) [54], limited support for this intervention exists due to a lack of evidence cited in the literature [53]. Because of this, placement of an FMS or colostomy should be decided individually and only recommended for functional impairments of the anus caused by thermal injuries.

### 3.6. Management of Frostbite

A less discussed but possibly devastating injury is frostbite injury of the genitalia. Though occurring less often than burns, frostbite of the genitals can also cause serious injury. Like burns, frostbite severity can be assessed by the degree of injury by examining the injury. First-degree frostbite consists of hyperemia with intact sensation and no blisters, and second-degree frostbite may present with fluid-filled blisters and edema. Third-degree frostbite may present as a sharp or throbbing pain and will contain hemorrhagic blisters. Lastly, fourth-degree frostbite will look like third-degree frostbite, but the skin may appear cyanotic and mottled due to deeper structures becoming frozen. Tissue loss can be expected in third-degree and fourth-degree frostbite.

Unfortunately, the incidence of frostbite of the penis and scrotum is not well assessed, so limited literature is available to describe specific management of frostbit genitalia. Risk factors for people who may develop frostbite have been well described and include dementia, mental illness, homelessness, old age, inexperience in new climates, alcohol/drug use, and the presence of extremity [55].

Patients may present with first-degree frostbite of the penis discussing symptoms of dysuria, numbness, erythema, and edema [56]. Though there are few reported cases in the literature describing frostbite of the perineum and genitals, there is a consensus that initial treatment of the mild frostbitten genital area includes rewarming the site in a warm bath (37–39 °C) [57]. Rewarming baths led to the resolution of symptoms in two patients with frostbite affecting the glans penis based on two recorded cases, though future research in this area could lead to more up-to-date recommendations [57,58,59]. While frostbite is less likely to impact the vulva [57], the management of severe frostbite in this area remains unclear due to a lack of literature on this topic. Overall, frostbite of the genitals can be successfully managed conservatively, much like burns and scalds of the perineum.

On the other hand, treatment of genital frostbite may involve more invasive interventions. For example, full-thickness frostbite of the penis and scrotum developed after a patient incorrectly used aerosol spray around his genitals, resulting in self-induced frostbite [60]. Treatment for this patient included topical silver sulfadiazine, trafermin spray to assist in granulation, and local wound care. Ultimately, the patient received split-thickness skin grafting and meshed split-thickness grafts for the penile shaft and scrotum, respectively, with successful skin graft adherence in both [60]. Though the case report does not discuss long-term follow-up and complications following management, they provide guidance about treatment options for severe cryogenic burns. In sum, extensive frostbite management of the perineum closely echoes the management of individuals with severe burns (including possible debridement, application of skin grafts, and use of skin flaps) and should be treated on an individual basis.

### 3.7. Long-Term Sequelae

Several considerations should be made to monitor outcomes in patients with severe burns after successful treatment of their genital burns. Specifically, attention should be focused on the quality of life of these patients following recovery. Beyond functional complications, some patients may experience a variety of psychological distress, psychosocial impairments, depression, and sexual dissatisfaction, after being treated for genital burns [61]. While long-term physical and psychological complications are expected in people with genital burns, there have been no large cohort studies analyzing current outcomes in patients with genital burns.

Functional complications are also common. Specifically, the most common complication for perineal burns is contracture formation, which are defined as tight, shortened scars that form on the deficit and may cause pain or discomfort for the patient [62]. In this instance, most scars are allowed to mature for one year before correcting the defect using multiple Z-plasties of the medial thigh, perineum and scrotal skin [3,62]. Following Z-plasties, patients have reduced pain, improved comfort, and a broader range of motion [63]. Though less likely, burns can also cause urethral slough and urethral stricture, typically managed by delayed urological reconstruction [64]. Ultimately, while several reports described functional outcomes of genital burns, less is known about the mental and social impacts that patients might encounter.

### 3.8. Future Directions

**Figure 1:**
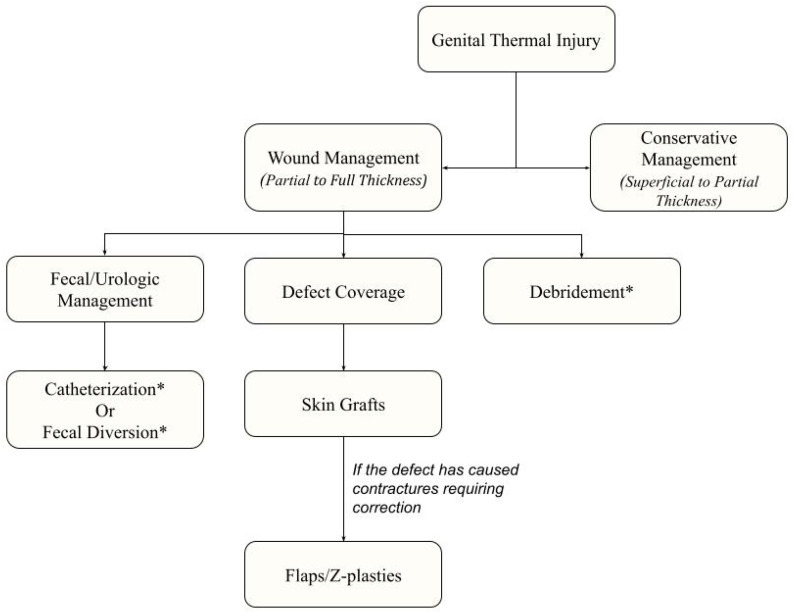
Conceptual Algorithm for Management of Genital Thermal Injuries of the Penis and Scrotum. Asterisks “*” indicates disagreement among the literature regarding the intervention.

This literature review has demonstrated that there is limited research investigating the optimal treatment of genital thermal injuries of the scrotum and penis (Figure 1). Future studies should be created to focus on the effectiveness of early vs. late surgical debridement of genital burns, determine specific criteria for determining when catheterization is needed, and monitor quality-of-life outcomes after patients recover from severe genital burns. As more research is gathered, we may one day be able to make more informed decisions regarding the management of genital burns.

## 4. Conclusions

Though a rare injury, perineal and genital thermal injuries disproportionately impact pediatric populations and may have long-lasting impacts on physical, sexual, and psychological functions. The gold standard for the initial management of genital injuries has involved a non-surgical, conservative approach, supported in the literature by high rates of injury resolution and lower long-term consequences. Delayed wound healing and severe burns are the primary indication for surgical intervention, and evidence supports limiting urinary catheterization for resuscitation purposes only to treat burns. Fecal management systems lack evidence for efficacy and should not be officially recommended. Multifaceted, long-term outcomes of patients with perineal burns are a future area for researchers to investigate.

## Data Availability

Not applicable.
